# Glass Powder Additive on Recycled Polypropylene Filaments: A Sustainable Material in 3D Printing

**DOI:** 10.3390/polym14010005

**Published:** 2021-12-21

**Authors:** Ruben Bayu Kristiawan, Boby Rusdyanto, Fitrian Imaduddin, Dody Ariawan

**Affiliations:** Mechanical Engineering Program, Faculty of Engineering, Universitas Sebelas Maret, Jl. Ir. Sutami No.36A, Kentingan, Surakarta 57126, Indonesia; yrbk.ruben@student.uns.ac.id (R.B.K.); bobyrusdyanto20@gmail.com (B.R.); dodyariawan@staff.uns.ac.id (D.A.)

**Keywords:** material extrusion, 3D printing, glass powder, composite filament, recycle polypropylene

## Abstract

This study aimed to characterize the effect of a glass powder additive on recycled polypropylene (rPP) materials from food packaging to be used as filaments in material extrusion (MEX) 3D printing applications. The composite filaments studied were rPP filaments with glass powder (GP) additive in the 2.5%, 5%, and 10% fractions. As a baseline, the filaments made of pure virgin PP and rPP without additive were used. The filament that has been successfully made is then printed into a tensile test specimen and an impact test to observe its mechanical properties. Fourier-transform infrared spectroscopy (FTIR) characterization was also carried out to determine the effect of chemical bonding and thermal characterization using thermogravimetric analysis (TGA) and differential scanning calorimetry (DSC). The results of FTIR characterization on the sample rPP + 10% do not show a typical peak shift of PP, but give rise to new peaks at wavenumbers of 1000 cm^−1^ (Si-O-Na), 890 cm^−1^ (Si-H) and 849 cm^−1^ (O-Si-O), which indicate the typical peaks of the glass constituent compounds. In the thermal characteristics, the addition of GP shows the improved stability of mass changes to heat and increases the melting temperature of rPP. The ultimate tensile strength and Young’s modulus for rPP-based specimens with 10% GP additive showed an increase of 38% and 42% compared to PP specimens. In addition to the improved mechanical strength, the addition of GP also reduces the bending deformation, which can be well controlled, and reduces curvature, which is a problem in semicrystalline polymer-based filaments.

## 1. Introduction

Since its discovery in 1954 and the start of commercial production by Montecatini in Ferrara in 1957, polypropylene (PP), a thermoplastic polymer, has become a successful commercial product [1,2]. Now, PP is the fastest-growing commodity. Consumer demand for PP is very high, making it one of the most significant plastic commodities. PP is generally used as a plastic packaging material due to its good chemical resistance and ease of processing methods. Unfortunately, PP packaging has a relatively short lifespan, making it one of the most found plastic waste, causing a harmful impact to the environment [3,4]. Recently, the growing trends of additive manufacturing (AM) methods present an opportunity to recycle thermoplastics to be used as AM feedstocks. Nearly 30 years since its conception, AM has gradually overcome specialized applications and revolutionized all kinds of practices in various manufacturing industries. AM offers a potential solution when conventional manufacturing reaches its technological limits. These include a high degree of design freedom, lightweight design, functional integration, and rapid prototyping. These advantages have led to AM being adopted since its beginning in the aerospace and defense industry, especially the U.S. military, for test parts in drones and satellites [5,6,7]. 

The principle of AM processing for polymer materials according to ASTM 52900 is divided into two categories, thermal reaction bonding and chemical reaction bonding. In general, thermal reaction bonding is more widely used in material extrusion (MEX) 3D printing, such as the fused deposition modeling (FDM) technique, while chemical reaction bonding is commonly used in stereolithography (SLA) 3D printing. Due to its affordability, MEX is one of the most widely used additive manufacturing technologies [8,9]. MEX provides flexibility in design, significant material savings, low cost, safety, customization, and individualism. As a result, MEX technology has partially replaced many conventional manufacturing processes and enabled new business models, products, and supply chains to evolve. The material used in MEX technology is mostly a thermoplastic polymer filament with a relatively low processing temperature. The MEX work by melting the filament using the heat obtained from the nozzle and shaping it to form a new shape. The nozzle extrudes and guides material in thin layers to produce structural elements layer by layer. Thermoplastic polymers such as acrylonitrile butadiene styrene (ABS) and polylactic acid (PLA) are widely used in MEX because of their low shrinkage and high stiffness. ABS has good durability and is often used in furniture, automotive, and other general industrial applications. At the same time, PLA is a thermoplastic with rigid, strong, and is easy to process characteristics, making it highly popular in AM applications. PLA can also be used as an implant material in the health sector [10,11,12]. 

The use of PP material in the MEX method is generally less common than PLA and ABS due to several challenging characteristics of PP in 3D printing [13,14,15,16]. For example, it quickly shrinks when it cools down, which leads to warping problems and fails the print. PP also only bonds with the PP material, limiting the bed selection. Moreover, it generally has lower tensile strength than PLA and ABS. Luckily, several studies have proved that the characteristics of PP can be changed in several ways, one of which is using additives as reinforcement [4,17,18,19,20]. The addition of reinforcement has been shown to increase the strength of PP and reduce its shrinkage properties. Some additive materials used as reinforcement, such as carbon black, glass, single wall carbon nanotubes (SWCNT), and graphene, are becoming quite popular for use [6,21,22,23,24].

In line with the effort to modify the PP characteristics, recycled PP (rPP) has also been investigated as one of the MEX material choices. The abundance of plastic waste is why choosing the rPP as the material for 3D printing might lead to a sustainable path. However, processing the rPP into an MEX filament is quite challenging as the rPP needs to undergo multiple heating cycles, which might degrade the polymer structure. The degradation, in particular, might change the characteristics of the polymer drastically so that the characteristics of the processed rPP will not be similar to the virgin PP anymore. Therefore, adding reinforcement additives to an rPP material will probably have different results from a virgin PP material [6,25,26,27]. 

The main objective of this paper is to investigate the mechanical characteristics of the rPP with glass powder additive and how it differs from the one without additive and the virgin PP material. The rPP is obtained and processed from the food packaging plastic waste made of Biaxially Oriented Polypropylene (BOPP). The glass powder is chosen in this study because, in some manner, it can be collected and processed from waste sources as well. Some studies also have shown that the addition of glass filler to PP filaments increases mechanical strength and reduces shrinkage. This work’s main contribution is in the area of new value creation in the rPP recycling process by demonstrating an alternative use of rPP in the additive manufacturing sector. The higher values of rPP recycling products in the additive manufacturing sector are expected to drive a new solution to reduce plastic waste.

## 2. Materials and Methods

### 2.1. Materials

#### 2.1.1. Post-Consumer Recycled Polypropylene Food Packaging

**Figure 1:**
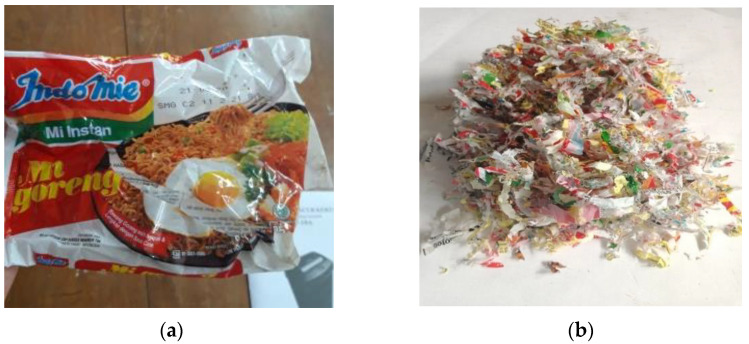
(**a**) Instant noodle food packaging. (**b**) Small pieces of instant noodle food packaging after shredding process.

The rPP used in this study is processed from instant noodle food packaging made of BOPP according to the specifications from the manufacturer PT. Indofood Sukses Makmur (Tbk), as shown in Figure 1a. The packaging was collected directly from several food vendors to minimize dirt and other waste contamination. A simple cleaning step using water is used before the plastic is shredded into smaller pieces, shown in Figure 1b.

#### 2.1.2. Polypropylene Filament

As a benchmark, a commercial polypropylene filament supplied by Kreafil is used with a diameter of 1.75 mm, with detailed specifications shown in Table 1 as follows:

#### 2.1.3. Glass Powder

**Figure 2:**
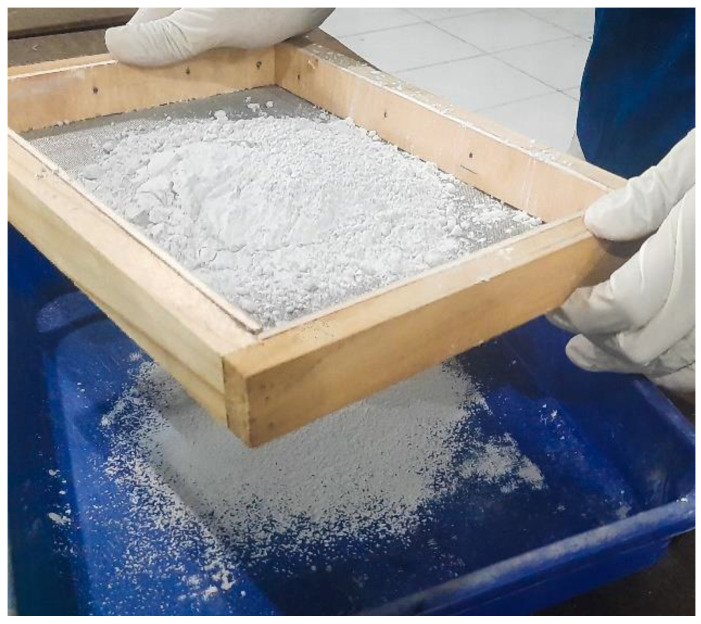
Filtering ground glass with a filter with a mesh size of 200.

Glass powder (GP) is used as an additive for reinforcement of recycled polypropylene filaments. Figure 2 is the GP used in this study that has less than 74 μm size that was prepared by filtering ground glass with a filter with a mesh size of 200.

### 2.2. Methodology

#### 2.2.1. Recycled Polypropylene Filament Fabrication

**Figure 3:**
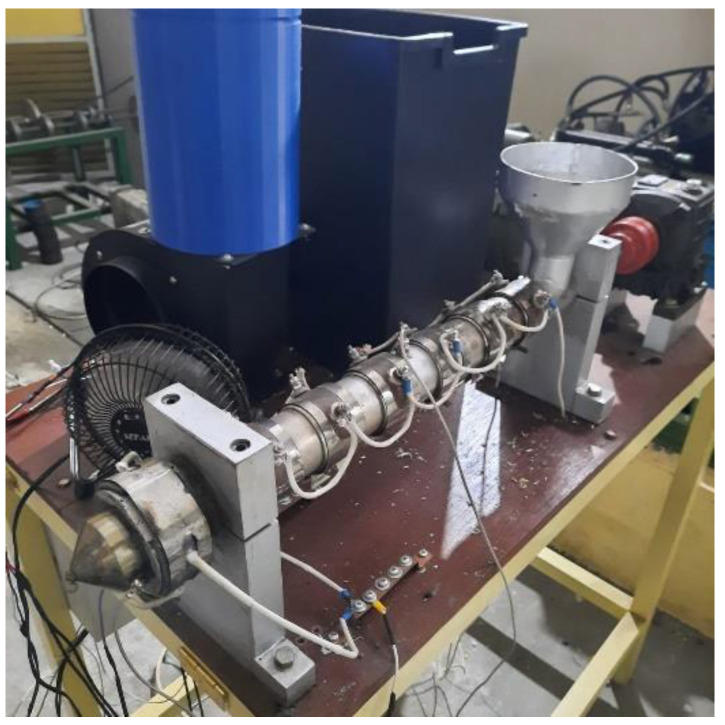
Single screw extruder machine.

The first process of treating food packaging waste is shredding the plastic sheet using an in-house shredder machine. In this machine, the plastic is cut into small sheets of 3–15 mm to ease the feeding process into the extruder machine. The second stage is the plastic extrusion process using a single screw extruder machine, shown in Figure 3. In the extrusion process, the temperature is set from 175–185 °C with a screw rotation speed of 24 RPM. The final process is the extrusion of the plastic compound into a 1 mm nozzle.

The filament exits the extruder nozzle in a hot semi-liquid compound. The screw push causes the filament size to expand and be slightly larger than the nozzle diameter. Then, the filament that comes out is cooled with a fan to anticipate the size of the filament shrinking when pulled by the spooler. The resulting Recycle PP filaments range in size from 1.5 to 2 mm.

Glass powder-reinforced rPP filaments were also processed by a similar method. However, before rPP is inserted into the hopper on the extruder machine, the chopped rPP plastic is mixed with glass powder before the mixing process is carried out using a mixer with a stirring duration of 5 min. The composition of the extruded material can be seen in Table 2.

#### 2.2.2. 3D Printing

The 3D printing machine is Creality Ender 3 with CURA software as a slicer for the CAD drawing. The initial trial of printing using conventional methods on heated printing platforms is unsuccessful due to lack of adhesion. A 1 mm thick polypropylene sheet is mounted on a heated glass printing platform and is used for all subsequent printing. The filament is printed at a nozzle temperature of 180–220 °C and bed temperature of 80 °C. The melted filament is pushed through a 1 mm nozzle at a speed of 20 mm per minute with a layer thickness of 0.32 mm. The information about the printing parameters of each type of filament can be seen in Table 3.

#### 2.2.3. Tensile Testing of 3D Printed Filament

**Figure 4:**
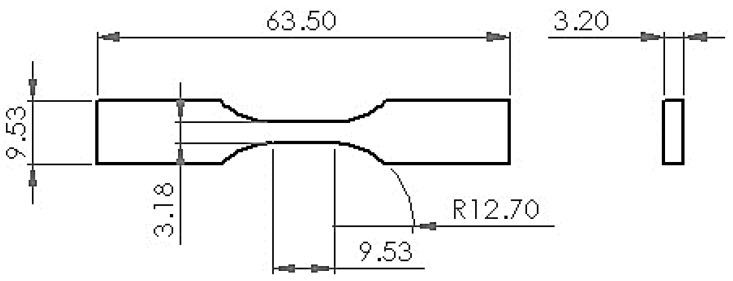
Tensile test specimen size for type 5 ASTM D638-14.

The filament tensile test specimens are printed with reference to the type 5 ASTM D638-14 standard as shown in Figure 4. The type 5 specimens were chosen because the material to be printed was only available in small quantities. The printed specimens were tested using a JTM-UTS510 tensile testing machine with a 10 mm/min loading speed.

#### 2.2.4. Impact Testing of 3D Printed Filament

**Figure 5:**
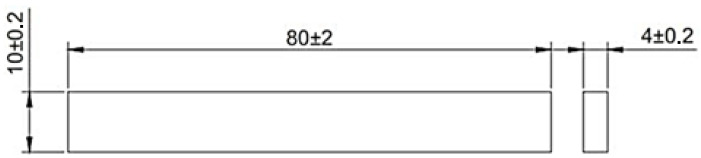
Impact test specimen size.

The impact specimen test was carried out using the Izod method, where the filament was molded based on the ASTM D256-10 standard as shown in Figure 5. The impact test equipment used is the Toyoseiki, Japan, which has an initial pendulum angle of 135 and a pendulum weight of 20.4274 N.

#### 2.2.5. Fourier-Transform Infrared Spectroscopy (FTIR)

The FTIR test is conducted using Cary 630 ATR-FTIR analyzer by Agilent, USA, to analyze the mixing effect on the extrusion method and 3d printing process with a spectral range of 500–4000 cm^−1^. The spectrums were recorded for pure PP, Packaged PP, and rPP to confirm the addition of glass powder to rPP. For each increase in wavelength on the x-axis, the corresponding transmittance value is plotted on the y-axis.

#### 2.2.6. Thermal Characterization

Thermogravimetric analysis (TGA) was used to analyze thermal stability and mass loss changes. Several samples were characterized to find the ratio of mass percentage reduction when the material was processed by being subjected to a working temperature at a heating rate of 10 °C/min, with the same sample used in the characterization of DSC.

Differential scanning calorimetry (DSC) was used to observe the melting behavior of PP, rPP, and rPP + 10% GP plastic packaging samples processed using Linseis STA PT 1600, Germany. Samples were obtained from pieces of plastic packaging and extruded filaments. The DSC sample was heated from 25 to 250 °C at a 10 °C/min rate.

## 3. Results

### 3.1. FTIR

**Figure 6:**
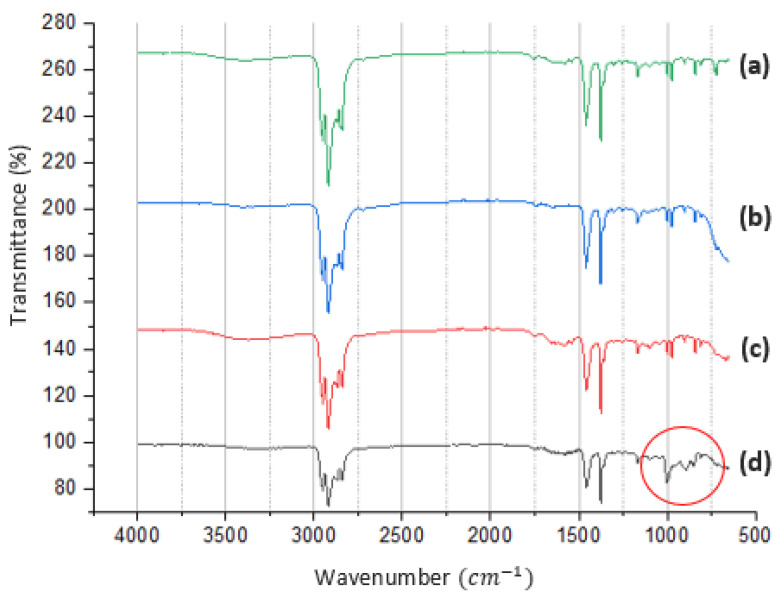
FTIR ATR spectra of (**a**) PP. (**b**) Food packaging plastic. (**c**) rPP; (**d**) rPP + 10%.

FTIR was performed on various samples to ensure that the packaging constituent compounds are PP and demonstrate the chemical reactions in the melting process. In this study, several materials were used, including pure PP, food packaging, rPP, and rPP + 10%. The functional groups in the material were analyzed using FTIR ATR. The results of the characterization of pure PP, food packaging, rPP, and rPP + 10% GP are shown in Figure 6.

The functional groups on pure PP material, food packaging, and rPP showed the same peak. The peaks formed include the wave number 2918 cm^−1^, which indicates the presence of C-H in CH_2_. The results are also supported by the company of a peak and 843 cm^−1^. In addition, the wavenumbers of 1459 cm^−1^ and 1377 cm^−1^ indicate the presence of a C-H group on CH_3_. At the length of the wavenumber of 933 cm^−1^ another peak also appears, indicating the presence of a C-C bond group. The material analysis results of rPP + 10% GP showed the same peaks as in rPP, with additional peaks formed at wavenumbers of 1000 cm^−1^ (Si-O-Na), 890 cm^−1^ (Si-H) and 849 cm^−1^ (O-Si-O). The occurrence of all peaks indicated is summarized in Table 4.

### 3.2. TGA and DSC

**Figure 7:**
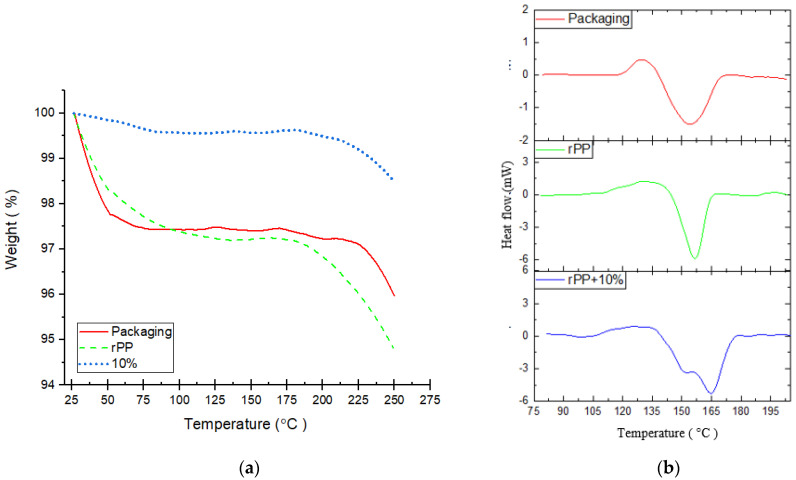
Thermal characterization of packaging plastic, rPP and rPP + 10%. (**a**) TGA; (**b**) DSC.

TGA and DSC were used to identify the thermal behavior of the materials that led to determining the suitable printing process parameters. In this case, the TGA test is monitored at a maximum temperature range of 250 °C. It was done by considering the purpose of the material as an MEX filament that is currently working with a maximum temperature of 250 °C. The three samples tested were primary samples, namely the raw packaging materials, the rPP, and the rPP + 10% GP. The results of the TGA test can be seen in Figure 7a.

From the TGA test results, the mass decrease in the initial phase occurs in the sample packaging until the temperature reaches 74 °C with a mass reduction of 2.5%. The following two steps of mass reduction were observed: a temperature of 169 °C with a mass reduction of 0.2% and the next phase at a temperature of 215 °C with a significant downward trend. The first phase in the rPP sample occurred up to a temperature of 97 °C with a mass reduction of 2.78%. Then, it is followed by a decomposition phase at a temperature of 173 °C. Different things happened to the rPP + 10% material; the mass decrease in the first phase occurred up to a temperature of 87 °C with a reduction of 0.44% mass. The decomposition phase of the sample rPP + 10% started at a temperature of 193 °C. Therefore, it can be seen that decomposition of the sample rPP + 10% is only 1.44% at a maximum temperature range of 250 °C, while the packaging is 3.99% and rPP is 6.16%.

The degree of crystallinity and melting properties were observed using DSC. It can be seen in Figure 7b. The three samples characterized by DSC showed some differences. Packaged samples have a melting temperature (Tm) similar to rPP, namely PP (155.24 °C) and rPP (156.58 °C). The crystalline temperature (Tc) in the two samples was not much different, namely PP (129 °C) and rPP (129.5 °C). Meanwhile, the rPP + 10% sample experienced a slight difference with the appearance of two Tm peaks at temperatures of 152 °C and 165 °C. It can also be seen that the Tc of the sample rPP + 10% is at a temperature of 131 °C. 

### 3.3. Tensile Test

**Figure 8:**
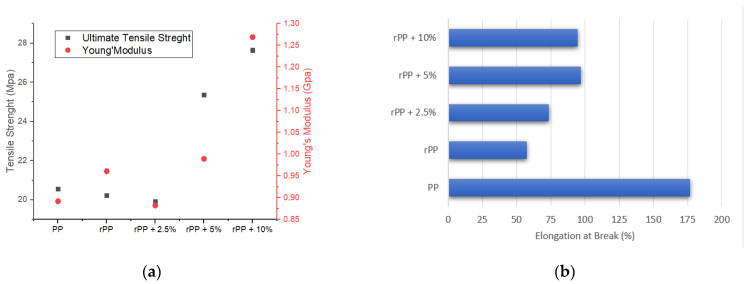
(**a**) Tensile strength and Young’s Modulus. (**b**) Elongation at break.

The initial mechanical behavior of the specimens was assessed using a tensile test. The tensile strength results are shown in Figure 8a. The results showed that the ultimate tensile strength values of PP (20.56 Mpa) and rPP (20.23 Mpa) and rPP + 2, 5% (19.91 Mpa) are not much different, while there are a significant increase in rPP + 5% (25.36 Mpa) and rPP + 10% (27.64 Mpa). Although the ultimate tensile strength difference is not significant, a slight difference was found in Young’s modulus of the PP, rPP, and rPP + 2.5% materials. The Young’s modulus of rPP (0.96 Gpa) was higher than PP (0.89 Gpa) and rPP + 2.5% (0.88 Gpa). Meanwhile, Young’s modulus of rPP + 5% (0.99 Gpa) and rPP + 10% (1.27 Gpa) are consistently higher than the results obtained by their counterparts.

Elastic properties of PP material with high elasticity, Therefore, this material does not break immediately after reaching UTS. Figure 8b shows how the elongation of the sample occurred when the tensile test was carried out. The figure stated that PP had the highest increase in elongation before experiencing failure, namely 176%. rPP has an elongation value of 57%. Figure 8b also shows that the addition of GP content increases the elongation value of rPP. The highest increase occurred in rPP + 5% material, which was 96.9%.

### 3.4. Impact Test

**Figure 9:**
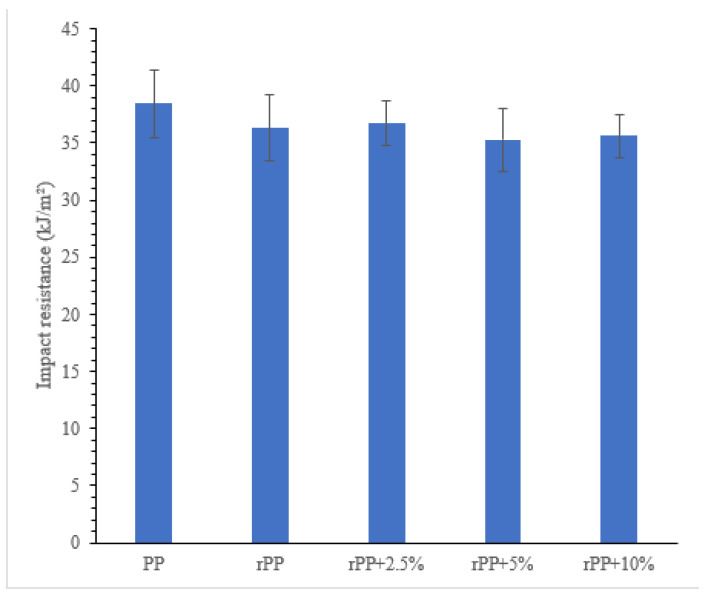
Impact test result.

Figure 9 shows the impact strength of each sample printed with the standard Izod impact test. It can be seen that the PP sample has the highest impact strength of 38.5 kJ/m^2^, followed by the rPP sample, which has an impact strength value of 36 kJ/m^2^, the same as the rPP + 2.5% sample. The addition of 5% and 10% GP percentages in the sample also showed a slight decrease in the impact strength. rPP + 5% and rPP + 10% have impact strengths of 35 kJ/m^2^.

## 4. Discussion

### 4.1. Filament Fabrication Analysis

**Figure 10:**
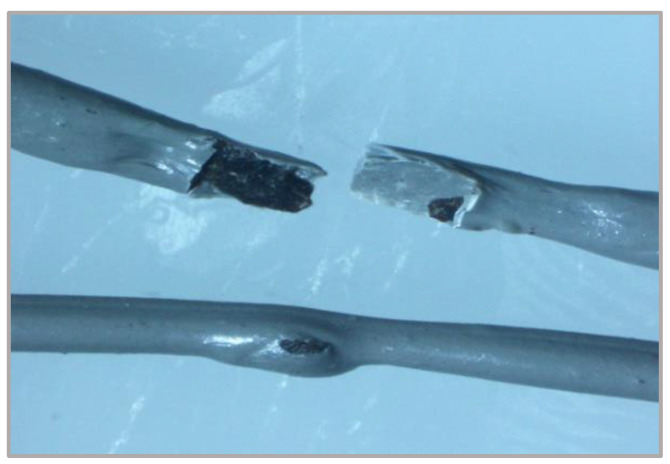
Impurities contained in the extruded filament.

In the rPP-based filament extrusion process, several problems were encountered; one of them is the presence of impurities in residues from packaged food components and other compounds that stick when the plastic packaging is in the trash. Impurities in the extrusion results resulted in unstable filament diameter, as shown in Figure 10, resulting in blockage of the extruder nozzle and breaking the extruded filament. The presence of these impurities is also a problem during the printing process because it can clog the print nozzle, which causes the filament to be pushed not smoothly so that the layer made by the MEX machine is not achieved correctly and results in defects in the results of the samples made.

**Figure 11:**
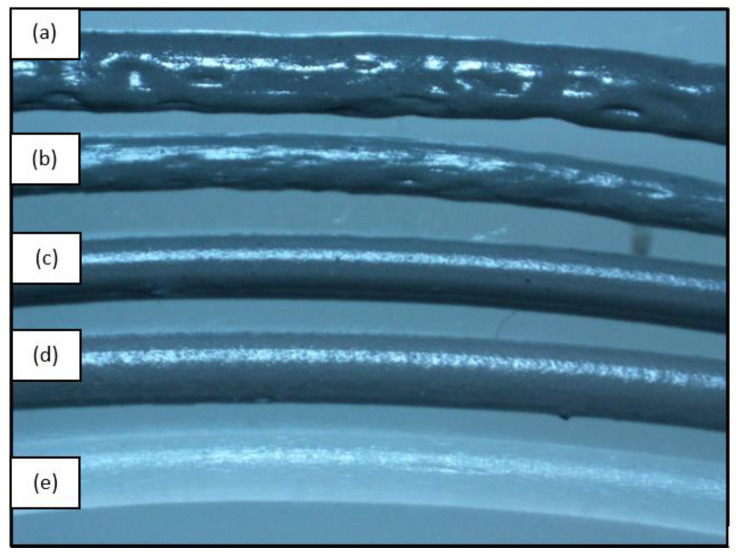
Extruded filament (**a**) rPP; (**b**) rPP + 2.5%; (**c**) rPP + 5%; (**d**) rPP + 10%; and (**e**) PP.

The filaments produced in the extrusion process have quite different characteristics. In rPP material, the surface is uneven, and there is much air trapped in the filament, which can be seen in Figure 11a. On the other hand, the addition of GP content to the rPP filament shows that the continuity of the diameter and shape of the filament can be achieved well. The higher the value of glass powder contained in the rPP filament, the better the continuity. As in Figure 11b, which is an rPP filament with the addition of 2.5% glass powder and followed by Figure 11c,d with 5% and 10% glass powder. This continuity was obtained because the heat dispersion given to the rPP material is better supported by the GP distribution in the rPP material. The diameter and shape of 10% rPP have an excellent approximation to the commercial PP filament used as a benchmark in this study [18,28,29,30].

Another characteristic change can be seen from the fading texture in the filament surface due to the addition of glass powder. In rPP filament, the surface is more glossy and has a brighter color. However, as the glass powder content increase, the surface color of the filament gets faded, and the texture becomes rough. Previous studies have reported similar effects of glass powder dispersion on the filament surface [31,32,33]. As the melting temperature of glass powder is higher than rPP, the glass powder mixture does not blend perfectly when the melt blending process with rPP results in a rough filament surface [17,27].

### 4.2. Printing Filament Analysis

The filament printing process is carried out with the nozzle temperature set in the melting temperature range until the material temperature begins to degrade in mass from the TGA characterization process using a temperature of 180–220 °C. However, the best printing results are obtained in the temperature range of 210–220 °C. In the PP filament printing process, which is the benchmark for this research, the printing process does not experience any problems in the printing process. However, there are some problems with rPP filament and rPP + GP filament during the printing process. Impurities present in the filament can become obstacles during the printing process. It can clog the nozzle of the 3D machine and cause the printing result to lose its constituent layers, and even stop the printing process. The impurities can also be related to the decrease in the mechanical strength of the printed sample.

**Figure 12:**
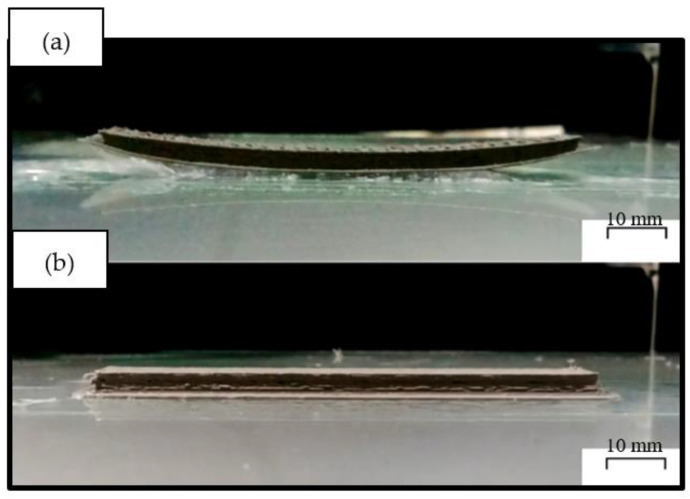
Results of printed samples of Izod impact specimens with rPP material: (**a**) rPP; (**b**) rPP + 10%.

Then, the problem occurs due to the inherent nature of PP, namely warpage caused by shrinkage that occurs during the material cooling process after the printing process. Semicrystalline polymers such as PP are susceptible to uncompensated volumetric shrinkage and contraction because the specific volumes of the melt and solid phases differ significantly [5,34,35]. In Figure 12, the Izod Impact specimen is printed with five specimens to guarantee good repeatability and the best printability. It is visible that the warp occurs in the rPP filament, where the initial layer was pulled to the center due to the material shrinkage. As the inorganic GP filler successfully inhibited the volumetric change of the polymer chains, the volumetric shrinkage decreased with increasing filler load and successfully reduced the warpage of the rPP specimens. PP neatly behaves like a thermal insulator, which delays the thermal equilibrium. Therefore, an inhomogeneous temperature distribution of the molded part is still present after the molding time.

On the other hand, glass-filled rPP is more thermally conductive and will approach thermal equilibrium faster than rPP. Thus, the higher thermal conductivity caused by GP incorporation leads to a more homogeneous temperature distribution in the fabricated specimen during molding. As a result, the internal/residual stresses of the material are also expected to be reduced, which explains the good dimensional accuracy [5,26,29,36].

### 4.3. Material Analysis

Materials were analyzed, starting from material characterization to testing to determine the strength of the material. Characterization starts from the FTIR test; pure PP, food packaging, and rPP showed the same peak. The peaks formed at these wavenumbers are the typical peaks of polypropylene material. In conclusion, the result indicates that recycled packaging has a similar primary material structure to PP [37,38,39,40,41]. The new peak at sample rPP + 10% describes the typical spectrum possessed by sodium silicate, which is the constituent of GP material, and it is concluded that the addition of glass powder material to rPP material has been successfully carried out. The addition of glass powder material did not cause a shift in the typical polypropylene peak, but gave rise to a new characteristic peak [32,39,41,42,43,44,45,46].

From the TGA test, it can be seen that the sample packaging and rPP experienced an apparent initial decrease where this phase is the drying phase or the loss of volatile compounds contained in the sample [2,47,48,49]. These results show that the volatile compounds in the packaging sample and rPP are much more, which can be caused because the packaging has not undergone an extrusion process that can reduce its volatile compounds. In contrast, rPP has reduced volatile compounds when the extrusion process is carried out. The rPP + 10% sample has better thermal stability, including in the initial phase. The addition of GP is concluded to increase the thermal stability of the rPP material. GP can distribute heat better in the extrusion process because it is more heat conductive, which causes better heat distribution to the extruded material and removes volatile compounds.

On the other hand, GP has a higher level of heat resistance than polypropylene as a base material [50,51,52,53,54,55,56]. In addition, when the degree of crystallinity and melting properties were observed using DSC, the addition of GP has signaled *Tm* to shift higher. A higher *Tm* value indicates the crystalline nature of the polymer, which concludes that rPP + 10% has a higher crystallinity level at room temperature than the raw packaging material and rPP. It can also be seen that the Tc of the sample rPP + 10% is higher than other samples [26,29,47,57,58,59].

In terms of mechanical strength, the addition of GP has succeeded in increasing the strength of the rPP material filled with GP with a grain size of 74 m. It was seen that the tensile strength and Young’s modulus increased significantly. In this assay process, GP managed to maintain the minimum critical length of the samples, as the polymer chain mobility was reduced due to the addition of rigid microspheres [6,18,22,27]. Interesting events also occur with strain in the GP-added rPP samples. The increase in tensile strain occurs along with the increase in the percentage of GP, although it is still far below pure PP. This shows the success of GP in distributing heat to the rPP material and causing the bonding between polymer chains to increase, and increase the adhesion of each layer of the sample mold [19,26].

In contrast to tensile strength, the results of which are affected by the weld yield of each layer, the impact properties are determined directly by the matrix, filler, and interface. Since the Izod specimen is molded, the strands are oriented perpendicular to the direction of impact. The results showed that the PP sample has the highest impact strength, followed by the rPP sample, similar to the rPP + 2.5% sample. The addition of 5% and 10% GP in the sample also showed a slight decrease in the impact strength, attributed to the GP particles acting as defects that initiate cracks. According to Griffith’s theory, the large aggregate gate is a weak point that lowers the stress required for the composite to fracture [22,60].

**Figure 13:**
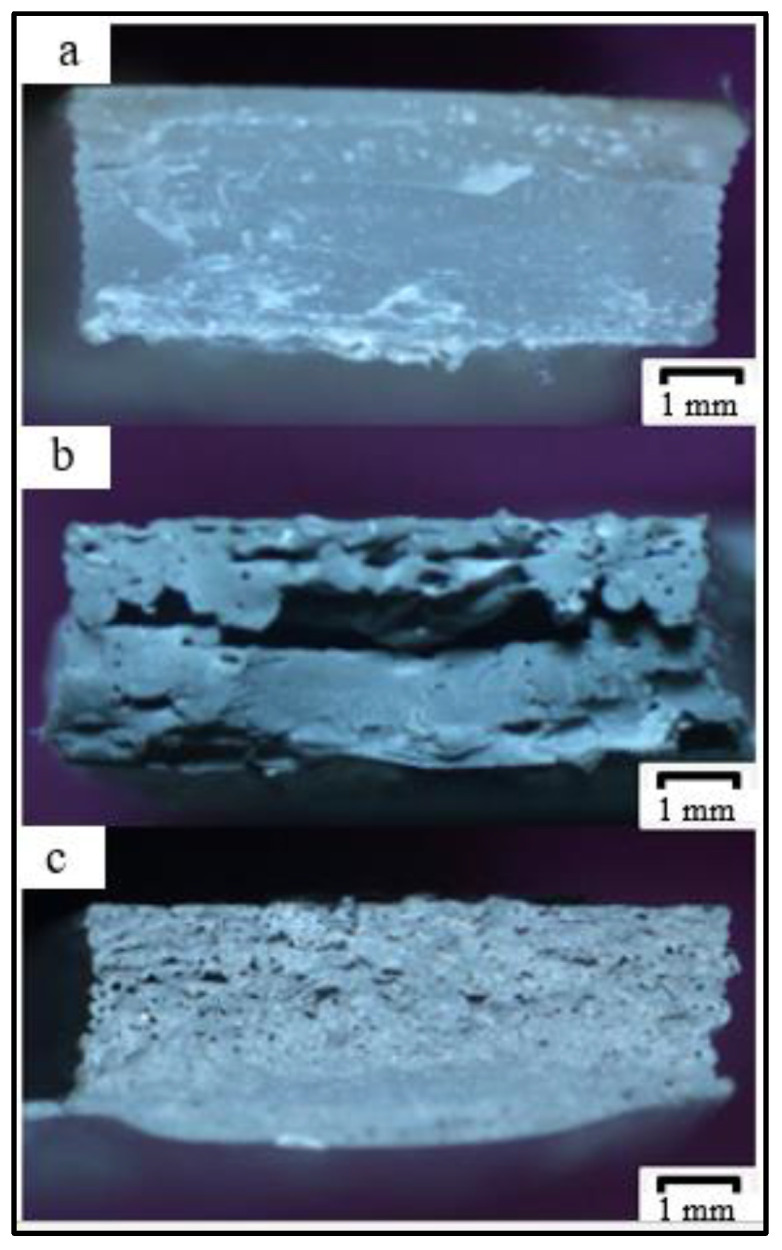
Impact test specimen fracture: (**a**) PP; (**b**) rPP; (**c**) rPP + 10%.

Figure 13 shows that the PP samples can be molded flawlessly due to the high yield strength and elongation at yield resulting from the homogeneous distribution of the layer material, its low crystallinity, and the correct nozzle temperature matched to the compound’s viscosity giving it high impact strength. In contrast, rPP material exhibits filament cracking in the fracture results due to its relatively lower strength and strain than PP. In addition, during the molding of the material, there was occasional nozzle clogging due to agglomeration of the impurity material mixed during the extrusion process. However, specimens can be printed to the desired size. From Figure 13, it is also clear how the shape of the rPP printed sample has a large crack in the middle. Meanwhile, rPP + 10% shows a small-scale porosity that spreads in many parts of the sample, which also causes the impact strength value to be lower than pure PP [61,62].

In the future, research will need to be focused on the filament fabrication process so that the shape of the filament can be manufactured continuously in the right shape and size with a homogeneous distribution of GP in the materials. Investigations of other additives also need to be investigated to compare the results of the addition of GP with other materials. In summary, this work proves that rPP can be a good choice for MEX, and, given its abundance, the use of rPP in MEX is a sustainable option that could lead to a new solution for the plastic waste problem.

## 5. Conclusions

This study demonstrated a successful combination of increasing the tensile strength and Young’s modulus of GP-filled rPP material. An increase in the breaking strain and a decrease in bending deformation occur with an increase of GP percentage. In addition to mechanical strength, the thermal stability of rPP also improves and slightly increases its Tm and Tc. This finding provides a solution to improve mechanical properties while optimizing dimensional control of printed rPP composites and providing added value to the material, which is waste. In the future, research will need to be carried out on the filament fabrication process, which aims to obtain continuous filaments in shape and size and is homogeneous as one of the requirements to make rPP filaments commercial.

## Figures and Tables

**Table 1:** Physical properties polypropylene filament supplied by Kreafil.

Physical Properties	
Density	0.90 g/cm^3^
Melting Point	136 °C/276.8 °F
Color	Natural
Diameter	1.75 ± 0.05 mm
Net Weight/roll	800 gr

**Table 2:** The composition and designation of the specimens.

Sample Designation	Composition
PP	Pure Polypropylene
rPP	Recycle plastic packaging
rPP + 2.5%	rPP + 2.5% Glass Powder
rPP + 5%	rPP + 5% Glass Powder
rPP + 10%	rPP + 10% Glass Powder

**Table 3:** Printing parameters for rPP, PP, and PLA filaments.

Parameters	Values
Nozzle diameter	1.0 mm
Layer thickness	0.32 mm
Infill degree	100%
Printing speed	20 mm/s
Bed temperature	80 °C with an insulating layer (rPP and PP)
Nozzle temperature	210 °C (rPP) 220 °C (rPP + Glass Powder) 210 °C (PP)

**Table 4:** FTIR analysis.

PP		Packaging		rPP		rPP + 10%		Vibrational Mode
WN	Intensity	WN	Intensity	WN	Intensity	WN	Intensity	
2868.2	69.2	2870.0	70.9	2868.2	73.8	2868.2	83.2	–C-H (CH_2_) stretching
1459.2	68.2	1459.3	70,6	1459.2	72,5	1459.3	80.1	C-O-H
1578.5	94.7	1638.2	96.4	1654.9	92.8	1656.8	95.1	C=C
1304.6	94.2	1304.6	94.4	1304.6	93.1	1304.6	94.5	C-H (-CH_3_)
900.2	94.9	900.2	94.5	900.2	93.6	900.2	93.3	C-C bond
840.5	89.6	842.4	90.2	840.5	88.4	840.5	90.0	CH_2_
						890.83	87.34	Si-H
						849.8	89.32	O-Si-O
						1000.8	82.4	Si-O-Na stretching vibration

## Data Availability

The data presented in this study are available on request from the corresponding author.
